# A First Pre-season Pollen Transport Climatology to Bavaria, Germany

**DOI:** 10.3389/falgy.2021.627863

**Published:** 2021-02-25

**Authors:** Annette Menzel, Homa Ghasemifard, Ye Yuan, Nicole Estrella

**Affiliations:** ^1^Department of Life Science Systems, TUM School of Life Sciences, Technical University of Munich (TUM), Freising, Germany; ^2^Institute for Advanced Study, Technical University of Munich (TUM), Garching, Germany

**Keywords:** flowering, HYSPLIT model, backward trajectory analysis, pollen season, pre-season transport

## Abstract

Climate impacts on the pollen season are well-described however less is known on how frequently atmospheric transport influences the start of the pollen season. Based on long-term phenological flowering and airborne pollen data (1987–2017) for six stations and seven taxa across Bavaria, Germany, we studied changes in the pollen season, compared pollen and flowering season start dates to determine pollen sources, and analyzed the likelihood of pollen transport by HYSPLIT back trajectories. Species advanced their pollen season more in early spring (e.g., *Corylus* and *Alnus* by up to 2 days yr^−1^) than in mid spring (*Betula, Fraxinus, Pinus*); *Poaceae* and *Artemisia* exhibited mixed trends in summer. Annual pollen sums mainly increased for *Corylus* and decreased for *Poaceae* and *Artemisia*. Start of pollen season trends largely deviated from flowering trends, especially for *Corylus* and *Alnus*. Transport phenomena, which rely on comparisons between flowering and pollen dates, were determined for 2005–2015 at three stations. Pre-season pollen was a common phenomenon: airborne pollen was predominantly observed earlier than flowering (median 17 days) and in general, in 63% of the cases (except for *Artemisia* and *Poaceae*, and the alpine location) the pollen sources were non-local (transported). In 54% (35%) of these cases, back trajectories confirmed (partly confirmed) the pre-season transport, only in 11% of the cases transport modeling failed to explain the records. Even within the main pollen season, 70% of pollen season start dates were linked to transport. At the alpine station, non-local pollen sources (both from outside Bavaria as well as Bavarian lowlands) predominated, in only 13% of these cases transport could not be confirmed by back trajectories. This prominent role of pollen transport has important implications for the length, the timing, and the severity of the allergenic pollen season, indicating only a weak dependency on flowering of local pollen sources.

## Introduction

Allergen pollen is a major health burden worldwide which is aggravated with anthropogenic climate change (1, 2): Spring warming leads to earlier flowering of species, thus an advance of the start of the pollen season; higher atmospheric CO_2_ concentrations reinforce flower—thus pollen—production; invasive species promoted by warming fill last gaps in the pollen calendar; and interactions with other air pollutants may intensify pollen allergenicity (3, 4). Indirect impacts on land use/land cover, e.g., by disturbances or adaptation in agricultural management (5), will further alter the spatial distribution and density of respective taxa. However, it is also likely that long- and medium-range transport is directly or indirectly altered by weather patterns and climate change, which would then in turn also affect the apparent pollen season at a specific site.

Lightweight pollen grains of anemophilous species are built to fly and after their release in large amounts, they can be dispersed and transported over several hundreds of kilometers, even into the Arctic (6). There is no generally applicable definition of medium- and long-range transport. Sofiev et al. (7) proposed transport with the wind, mixing inside the atmospheric boundary layer as well as dry and wet removal at the regional scale as key processes for medium-range transport, and dispersion with synoptic-scale wind plus exchange between the boundary layer and the free troposphere as key processes for long-range transport.

But the central question is of how to recognize transported pollen at the receptor site. Clear attribution of captured pollen to transport is only possible if e.g., species of these (exotic) taxa are not present in the vicinity of a station or for episodes with pollen concentrations larger than a predefined threshold mirroring local production volumes [see as example (8) or (9)]. Alternatively, also events outside the main pollen season of a species, i.e., pre-season and/or post-season events can be assigned to transport events [e.g., (10, 11)] or simultaneous high peaks at several stations [e.g., (12)]. Sometimes increased night-time concentrations may point to regional-scale transport (13). Such identified pollen can be delineated to sources or footprint areas by backward air mass trajectories e.g., from HYSPLIT [e.g., (11, 14, 15)]. Equally numerical atmospheric pollen dispersion models such as COSMO-ART (16) or SILAM [e.g., (8)] can assist in simulating (forward) pollen transport to be compared with measured atmospheric concentrations at selected sites or be used more sophistically for source apportionment [inverse problem, e.g., (17)].

Many papers have been published in recent years on when and how pollen is transported, often on *Ambrosia* with its pollen grains being small in diameter and limited source regions. Studies report *Ambrosia* pollen transport from Eastern Europe to Italy (12), from Western Europe to Hungary (18), from Central and southern Europe to the UK and the Netherlands (15), or from southerly directions such Ukraine to Poland (9, 19, 20). Similar transport features have been reported for *Betula* or *Alnus*, e.g., from Poland and Germany to Denmark (10) or Lithuania (17) and from Germany, the Netherlands, and Belgium to UK and Poland (21) or within Spain (22). However, only a few studies have completed the step from single case studies to the quantification of events and even to transported pollen amounts. Veriankaite et al. (17) identified 24 birch transport events in 4 years in Lithuania with highly variable spatial and temporal patterns. For the “Yellow dust episode” in Finland in spring 1991, Franzén et al. (23) provided an estimate of transported pollen amounts per cm^2^. Besides trajectories and dispersion models, pollen transport has also been linked to persistent wind directions (24) and atmospheric circulation patterns, e.g., highest pollen concentrations in Poland are registered for warm, sunny, and dry anticyclonic circulation types with advection from the SW (25). However, for Germany, less is known about the magnitude or extent of pollen transport.

To confirm or even validate pollen transport as assessed by the above mentioned methods, more data is needed, e.g., for a proper analysis according to Hernandez-Ceballos et al. (26) information on both pollen source locations and local tree flowering phenology. Newer studies even use satellite-based remote sensing data such as NDVI or EVI as proxies for flowering times (27). Quite interestingly Skjøth et al. (28) reported that the production of sources maps was sensitive to the type of underlying land cover data and their richness of detail e.g., in woodlands. In case that species within pollen taxa are morphologically not distinguishable, novel molecular analyses using PCR have been implemented to identify possible sources of long-distance transport, e.g., *Juniperus* pollen from Texas as part of the *Cupressaceae* pollen measured in Canada (29).

The potential consequences of this long-range pollen transport for practical applications are manifold. The main problem might be that the actual pollen concentration will be less dependent on local conditions and therefore local phenological data will not represent the actual pollen season [e.g., (30, 31)] complicating pollen forecast. Pollen transport might deliver more pollen, also from non-native or alien species, to a measurement site and/or will prolong the local pollen season and render it more variable at the end. E.g., Sofiev (32) quantified by a 35-yr SILAM re-analysi for Europe that transport accounted for 10–20% and 20–40% of the inter-annual variability in the seasonal birch and grass pollen index, respectively. Pollen transport is also seen as the main reason for experiencing additional pollen loads during the night-time (13), a time of the day which is recommended to allergic patients for airing their flats.

Based on long-term airborne pollen data of seven taxa and phenological observations of the start of flowering in Bavaria, SE Germany, we aimed at answering the following research questions:

1. Which are the apparent changes in key parameters of the pollen season in the last three decades and are changes in the start of the pollen season consistent between airborne and ground phenological observations?
2. How closely do flowering and start of pollen season dates correspond depending on pollen station and taxa?
3. How frequent are (long-range) pre-season pollen events and are these attributable to transport events by HYSPLIT back trajectories?

## Materials and Methods

The study was carried out in the free state of Bavaria, the federal state in the south-eastern part of Germany with four major landscape types: the Bavarian Alps in the south toward Austria and Switzerland, the Alpine foothills or Foreland with many lakes south of Munich, the eastern Bavarian central mountains toward the Czech Republic and in the west/north-west the plateaus of the Swabia and Frankenalb. 83% of its area is under agricultural and forest use (47% agriculture, 36% forests) (33).

### Pollen Data

**Figure 1:**
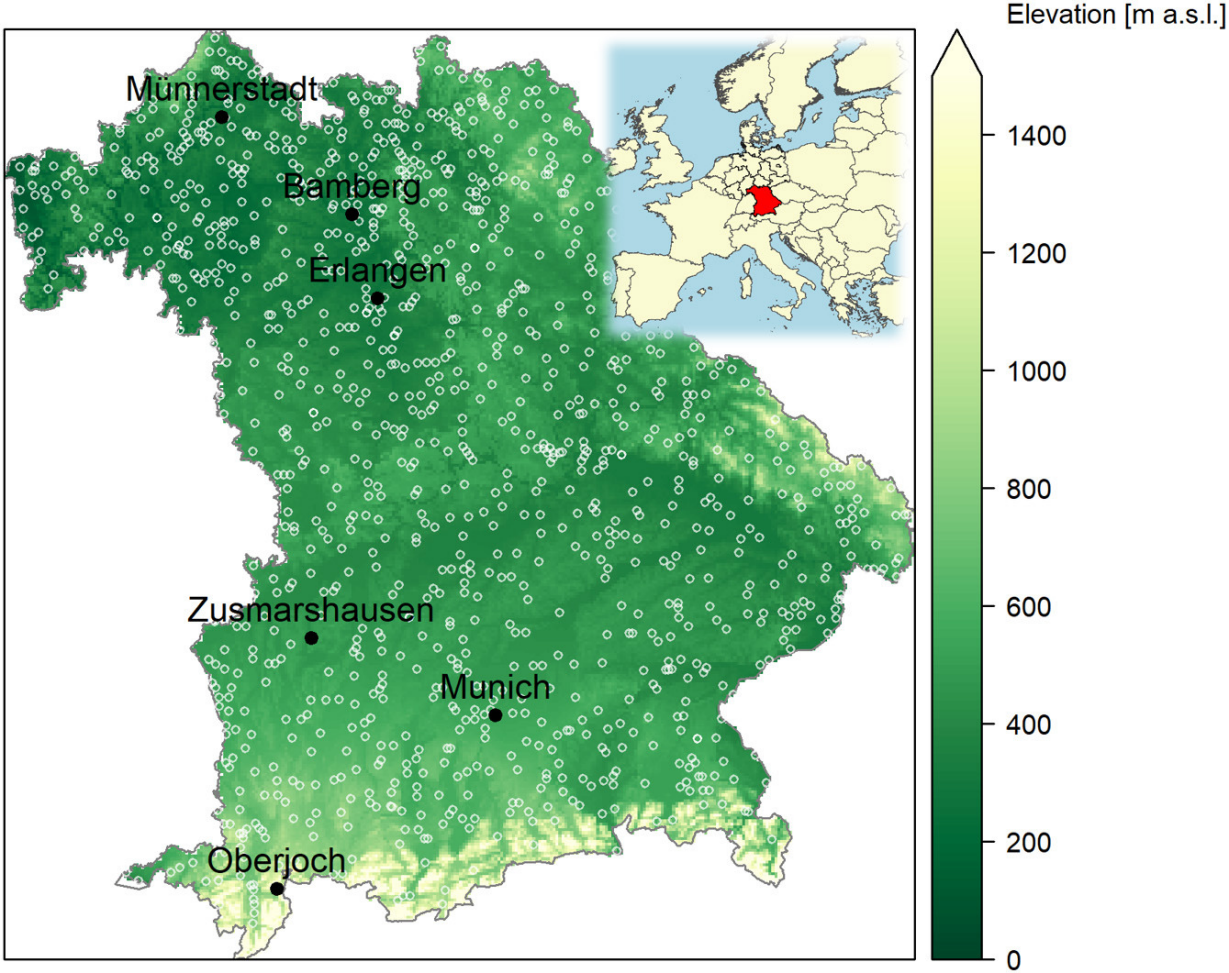
Phenological sites (white open circles) with flowering observations of the German Meteorological Service (DWD) and six sites (black filled circles) with long-term pollen monitoring of the German Pollen Information Service (PID) used in this study (background digital terrain model grid and state border from GeoBasis-DE/BKG 2020). The inset map of Europe shows the position of Bavaria as Germany's most southeastern federal state.

Daily atmospheric concentrations of allergenic pollen (pollen grains m^−3^) were available for six stations across Bavaria and a common time period of 1987 to 2017 (Figure 1, for further pollen site information see Supplementary Table 1). The station Oberjoch is alpine, while Munich (official pollen station name München) and Zusmarshausen are situated in the north of the alpine foothills, and Münnerstadt, Bamberg, Erlangen are in the warmer, north-western plateau region. Pollen monitoring was conducted with Hirst-type volumetric pollen traps, which started on average on Feb 8th (median Feb 5th). The data were collected, quality checked, and provided by the German Pollen Information Service (Stiftung Deutscher Polleninformationsdienst, PID). Details on the pollen monitoring can be found in Werchan et al. (34). We extracted seven pollen taxa (*Alnus, Artemisia, Betula, Corylus, Fraxinus, Pinus*, and *Poaceae*) for which corresponding flowering dates were available (see section Phenological Data).

For each station, start and end dates of the respective pollen seasons (SOS_P_, EOS_P_) were determined by the percentage method [see (35, 36)], by which the earliest and latest annual 2.5% of the pollen concentrations were eliminated. In addition, annual peak dates (PEAK_P_) and annual pollen sums (SUM_P_, or annual pollen integrals) were also derived. Supplementary Figure 1 allows a quick overview on basic descriptive statistics of the pollen seasons by the percentage method, which is recommended by Bastl et al. (37). For comparison, four other methods also implemented in the AeRobiology R package (38), namely the logistic method (39), the moving method (38), the clinical method (40) as well as the grains method (41), were equally applied for determining linear trends in start, peak, end of the pollen season as well as annual pollen sums. As there was a small residual risk that pollen monitoring was not started in time to always capture the start of the pollen season especially for *Alnus* and *Corylus*, we did a sensitivity analysis on SOS_P_ trends with two alternatively filtered data sets. We discarded SOS_P_ dates if the first day of operation already had pollen concentrations larger than four pollen grains m^−3^ and secondly, if the first day of operation was <10 days earlier than local flowering (see section Phenological Data and Supplementary Figure 5). For all five methods, we relied on the default setting of the AeRobiology R package of “lineal” imputation of missing values.

Daily pollen concentrations at SOS_P_ were classified according to Galán et al. (42), de Weger et al. (43), and Deutscher Wetterdienst (44) into intensity levels in order to assess the clinical relevance of long distance transport events.

### Phenological Data

Phenological data were retrieved from the German Meteorological Service (DWD) who is operating a volunteer network across Germany with about 1,200 sites in 2015 and only ~200 in Bavaria in 2017 (see Figure 1). We selected the phenological phases beginning of flowering of *Artemisia vulgaris* L. (common mugwort), *Alnus glutinosa* (L.) Gaertn. (European alder), *Betula pendula* Roth (European white birch), *Corylus avellana* L. (Common hazel), *Fraxinus excelsior* L. (European ash), *Pinus sylvestris* L. (Scots pine) as well as full flowering dates of two early grass species, *Dactylis glomerata* L. (Cock's foot or orchard grass) and *Alopecurus pratensis* L. (Meadow foxtail) for the period 1987–2017. No additional data filtering or quality assurance was performed since detailed observation instructions and automated data quality controls had been applied by DWD (45–47). *Artemisia, Dactylis* and *Alopecurus* are observed in plant communities of a site, for the latter two preferably on agricultural land. Flowering observations on the woody species are reported for a normal grown specimen at normal locations (microsites), preferably not in gardens and not on cultivated and garden varieties (45).

Annual phenological data were spatially interpolated following the proposed routine by DWD [(48, 49), Wolfgang Janssen, personal communication]. First Germany was divided into 30 overlapping circles of 1.95 degrees, then within each circle phenological onset dates (DOY) were regionally modeled by a multiple linear regression on elevation (h), longitude (lon) and latitude (lat) as independent variables (Equation 1).


(1) $$\begin{array}{r} DOY=a_{0}+a_{1}\;*\;h+a_{2}\;*\;lon+a_{3}\;*\;lat \end{array}$$

The corresponding regional regression coefficients were assigned to the respective center point of the circle and then spatially interpolated using inverse distance weighting (IDW) from the nearest two to four circles. For all 1 km^2^ pixels DOY were modeled based on geographical information and interpolated regression coefficients of the multiple linear regression model of equation 1. For all 70,609 1 km^2^ pixels, trends by linear regressions for the period 1987–2017 were calculated as baseline flowering trends in Bavaria. For the six pixels of the pollen stations, the respective annual onset dates (SOS_F_), as well as temporal trends, were extracted.

### Classification of Pre-season Transport Events by Local Phenological Data

We assumed that during the main pollen season of a specific pollen type it is not possible to disentangle local and transported contributions, but that the start of emissions from local sources should be well-mirrored by the interpolated respective start of flowering dates of DWD. However, one had to take into account inherent uncertainties in the interpolated flowering dates [RMSE of up to 10 days according to (50)], phenological variations with micro- and meso-climate in the study area and pollen emission amounts depending on the actual land use.

Thus, we defined pollen (SOS_P_) recorded <10 days from the interpolated flowering date at its 1 km^2^ pixel (SOS_F_) still as local and not necessarily as erroneous. Consequently, pollen season start dates equal to or more than 10 days earlier than the corresponding flowering dates at a station were considered as pre-season transport. Pollen season start dates equal to or more than 10 days later than local flowering hinted to either faulty pollen recording or lack of corresponding local sources (see classification rules in Table 1A). In addition we flagged trap operation starting dates after local flowering and discarded a few events which had more than ~20% of missing daily data in the pollen season, theoretically affecting the accuracy of the determined SOS_P_ dates. However, trends in pollen season start dates were largely unaffected by imputation of missing data as compared to raw data (see Supplementary Figure 5).

**Table 1:** Classification rules for pollen transport phenomena in Bavaria based on the start of pollen season dates (SOS_P_) for three stations (E Erlangen, M Munich, O Oberjoch) as compared to (A) start of local flowering (SOS_F_) of respective species in their 1 km^2^ surrounding and to (B) earliest flowering dates (SOS_T_) along 72 h back trajectories started on SOS_P_ as well as on 1 and 2 days prior to it (see section Likelihood of Transport Based on Back Trajectories).

**A Comparison of on-site flowering and pollen start dates (SOS**_**F**_ **, SOS**_**P**_ **)**			
**On site SOS_**P**_ - SOS_**F**_**	**Across stations SOS_**P**_**	**Likely pollen source**	**Type of transport**
(–∞,−10] days	O: SOS_P−O_ ≤ SOS_F−M/E_ −10 days	Pre-season transport	Long-range from outside Bavaria to O
	|SOS_P_ - SOS_P−other_| <3 days		Long-range
	Otherwise		Undefined range
(−10, 10) days		Local	Within-season transport not TBD
[10, +∞) days		No local sources	Transport unlikely
		Faulty pollen data	
**B Assessment based on SOS**_**F**_**, SOS**_**P**_ **as compared to flowering dates along trajectories (SOS**_**T**_**)**			
**72 h back-trajectories**		**Likelihood of transport**	
SOS_T_ ≤ SOS_P_		confirmed if at least on 1 out of 3 days	
SOS_P_ < SOS_T_ ≤ SOS_F_		partly confirmed if at least on 1 out of 3 days	
SOS_T_ > SOS_F_		rejected	

We applied this classification to the pollen data 1987–2017 of the stations Erlangen, Munich, and Oberjoch due to their superior data quality (see results Start of the Pollen and the Respective Flowering Season). For the high altitude station Oberjoch (see Supplementary Table 1), considerable local pollen sources should be present only for the taxa *Pinus* and *Poaceae*. Thus, for the other pollen taxa, generally certain sorts of transport phenomena were assumed and assigned to non-Bavarian sources if SOS_P_ was more than 10 days earlier than SOS_F_ at Munich and Erlangen. In general, similar SOS_P_ dates across stations (of not more than 2 days difference) also pointed to long-range transport events [(12); see Table 1A]. Post-season transport could not be identified in an equal manner since the end of flowering dates are not observed in the phenological program of DWD.

We compared the daily pollen concentrations at the start of pollen season dates (SOS_P_) and compared them for two likely pollen sources (see Table 1A), i.e., pre-season transport and local sources per pollen taxon and site. We used two sided *t*-tests for testing the significance of differences.

### HYSPLIT Transport Modeling

The HYSPLIT (hybrid single-particle Lagrangian integrated trajectory) model was applied to study air transport and characterize potential pollen source regions (51, 52). The ERA5 dataset of the European Centre for Medium-Range Weather Forecasts (ECMWF) provided hourly forecast weather data at a 30-km resolution for the necessary underlying meteorological model. ERA5 data were downloaded from the Copernicus Climate Change Service (C3S)/Climate Data Store (CDS) and, for earlier tests, from ECWMF MARS. They were retrieved and converted to ARL, the format required by HYSPLIT, using a technically customized version of get_era5_cds.py and era52arl.f publicly provided by A. Crawford (NOAA/ARL) at https://github.com/amcz/hysplit_metdata. The 72 h HYSPLIT back trajectories were run at 3 h intervals from 00:00 to 21:00 h local time (LT) for the pollen sites for the period 2005 until 2015 due to computational restrictions. The backward trajectory calculations were started at an altitude of 500 m above ground level. We plotted across Europe the respective eight back-trajectories per day from Erlangen, Munich, and Oberjoch for all SOS_P_ dates as well as for the 2 days prior and after SOS_P_.

### Likelihood of Transport Based on Back Trajectories

The likelihood of pollen transport by HYSPLIT trajectories was assessed for all pollen taxa based on the earliest respective start of flowering dates (SOS_T_) in the area covered by the 72 h back trajectories started at SOS_P_ as well as 1 and 2 days prior to SOS_P_ (see section HYSPLIT Transport Modeling). Taxa related species distributions following Caudullo et al. (53), Sofiev (32), and Holm et al. (54) (see Supplementary Figure 2) provided solid evidence for a complete pan-European distribution of each of the studied pollen taxa, except for parts of Spain and Portugal where *Alnus, Betula*, and *Corylus* pollen sources, of Italy where *Betula* and *Pinus* sources and of SE Europe where *Pinus* sources might be lacking. Since exact flowering dates for this large area were not available through the PEP725 database (55), we referred to the general movement of seasons throughout Europe (56): In early spring the green wave progresses from WSW to ENE, then from SW to NE and finally from SSW to NNE in late spring. In summer the movement is more south to north directed. Consequently, the annual start of flowering dates of Europe were extrapolated from the respective SOS_F_ dates of Erlangen and Munich (for Oberjoch we used the mean SOS_F_ of Erlangen and Munich). Extrapolation was made based on these season-specific speeds and directions of the green wave published (see Supplementary Table 2).

Then, the likelihood of pollen transport was assessed as medium if SOS_T_ was earlier or equal as SOS_F_ but later than SOS_P_ at the pollen receptor site, as high if SOS_T_ was even earlier or equal as SOS_P_, and in all other cases as zero (trajectories only over areas with later flowering dates than at the receptor site). The overall transport was assumed to be confirmed (partly confirmed) if at least on 1 out of 3 days (SOS_P_ and 1 and 2 days earlier) it was classified as high (medium) (Table 1B).

### Software

Analyses were performed using R statistical software (57) together with AeRobiology package (38). The backward trajectories were processed using the openair package (58) within R.

## Results

### Pollen Season Changes Across Time

**Figure 2:**
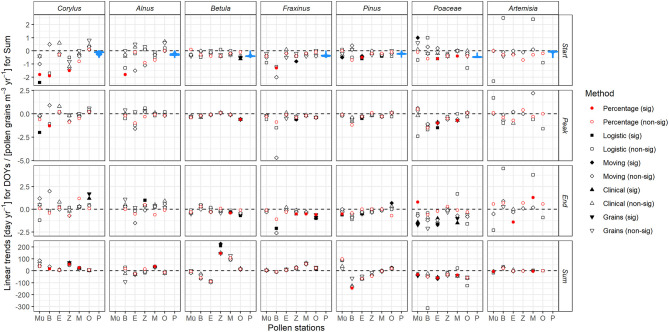
Changes in key parameters of the pollen season for seven selected taxa (column ordered by season from left to right) over 1987–2017 at six stations in Bavaria (from north to south Mü, Münnerstadt; B, Bamberg; E, Erlangen; Z, Zusmarshausen; M, Munich; O, Oberjoch). Values are slopes of linear regressions for start, peak and end of the pollen season (days yr^−1^) and for annual sums of pollen grains (m^−3^ yr^−1^) derived from five methods in the AeRobiology package (see section Pollen Data), red dots are for the percentage method used in the following. P shows as blue violin plots the slopes of linear regressions of interpolated flowering observations (SOS_F_) at all 1 km^2^ pixels across Bavaria. Significance tested at the *p* < 0.05 level.

Clear changes in key parameters of the pollen season were identified for the seven taxa across the six pollen stations (Figure 2), however only ~17% of the changes in the start, peak and end of the pollen seasons and ~27% of the changes in annual pollen sums, as assessed by the percentage method, were significant.

The early flowering taxa *Corylus* and *Alnus* mostly advanced their start and peak of season dates by up to 2 days year^−1^. Taxa flowering in mid spring such as *Betula, Fraxinus*, and *Pinus* advanced their start and peak of season dates less, by ~ up to 0.5 days year^−1^. Trends at the pollen station Bamberg were sometimes considerably stronger. *Poaceae* and *Artemisia*, flowering in late spring and full summer, exhibited uneven, i.e., advancing and delaying, trends with the exception of *Poaceae* peak dates strongly advancing in Munich, Erlangen and Bamberg. End of season dates were generally mostly unchanged. The choice of alternative approaches to the percentage method had no noticeable effect on the trends of key parameters of the pollen season (Figure 2) except SOS_P_ of *Corylus* and *Alnus* which were stronger at some stations and EOS_P_ which were weaker than by the alternative methods.

We therefore tested the robustness of SOS_P_ trends by the percentage method against possibly too late start of pollen monitoring after winter by two alternative data filtering methods. However, the results (see Supplementary Figure 5) indicated that only for the two early pollen taxa, namely *Corylus* and *Alnus*, trends slightly differed, but the main findings as described above were unaffected. Equally, we checked the effect of “lineal” interpolation of missing data during the pollen season on SOS_P_ trends; however, for most cases the differences between raw and imputed data were small or none except two cases (*Alnus* and *Artemisia* at Zusmarshausen).

Annual pollen sums (often significantly) increased across all stations for *Corylus* and decreased at all stations for *Poaceae* and *Artemisia* (the strong positive trend at Bamberg is due to a single outlier, see Supplementary Figure 3). We observed mixed and often insignificant changes in annual pollen sums for *Alnus, Betula*, and *Fraxinus*; however values for these three taxa at the southern stations (Zusmarshausen, Munich, and Oberjoch) were predominantly increasing, whereas for *Pinus* we derived more decreasing than increasing ones.

Trends largely deviating from the other stations mostly resulted from single year outliers at the start or end of the time series (see examples for *Artemisia* annual pollen at Bamberg and start of the pollen season at Zusmarshausen, Supplementary Figure 3).

### Start of the Pollen and the Respective Flowering Season

The second method to derive trends in the start of the flowering season was via year-wise interpolated phenological observations of the DWD. We summarized the 70,609 results across all 1 km^2^ pixels in Bavaria for each of the seven taxa, shown as violin density plots in Figure 2. Interestingly, the start of pollen season trends at the six stations were often outside the tails of the violin plots, thus largely deviating from the observed flowering trends, especially in the case of *Corylus* and *Alnus*, the early flowering species. Start of pollen season trends largely deviating from general flowering trends were found at Bamberg (more negative for *Corylus, Alnus, Pinus, Fraxinus*, more positive for *Artemisia*), equally at Münnerstadt (more negative for *Corylus*, more positive for *Poaceae*) and Zusmarshausen (more negative for *Corylus, Alnus, Artemisia*).

**Figure 3:**
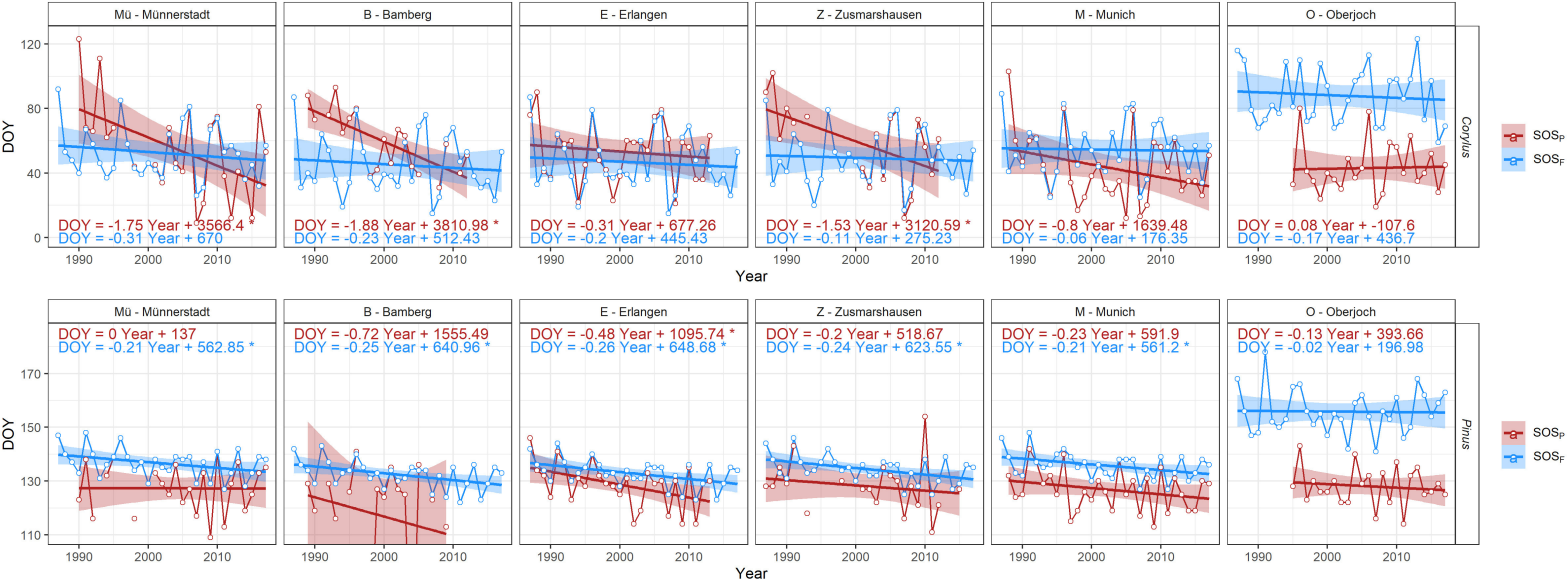
Time series and their linear trends of start of the pollen season (red) and of start of flowering (blue) for *Corylus*/*Corylus avellana* and *Pinus*/*Pinus sylvestris*, respectively, at six stations in Bavaria (ordered from north to south). Onset days are given as DOY (day of the year on the y-axis). The slopes of the trends and their significance (^*^p < 0.05) are given in Figure 2.

A closer look at the flowering and start of the pollen season time series revealed that pollen time series at these stations were considerably different from the corresponding flowering ones (Figure 3). Especially missing data for some years as well as failing procedures to determine the key parameters of the pollen season in the R package AeRobiology resulted in gaps in the time series and may have caused these pronounced differences, more prominent at the stations Münnerstadt, Bamberg, and Zusmarshausen. Time series and trends of flowering dates and start of pollen season dates at Oberjoch were comparable, however, the start of the pollen season was consistently earlier than the interpolated flowering dates due to the high elevation of this pollen station (see Supplementary Table 1). We, therefore, decided to study long-range transport, which mostly relies on the comparison of flowering and pollen dates, for the remaining three stations (Erlangen, Munich, Oberjoch). Supplementary Figure 4 shows in this respect two examples of strong outliers for the end of the *Betula* pollen season at Bamberg and Zusmarshausen.

### Pre-season Transport Events

Differences between pollen and flowering start dates at Munich and Oberjoch (2005–2015) and Erlangen (2005–2012) ranged between −88 and 53 days (Table 2). However, out of these 209 station ^*^ year ^*^ taxa combinations corresponding to the trajectory study (see section Transportation Modeled by HYSPLIT), 14 cases were not considered as reliable, eight of them related to *Artemisia* where the interpolated flowering dates seemed less robust (SOS_F_ at Oberjoch earlier than in the lowland) and six to *Corylus, Alnus*, and *Poaceae* at Erlangen where pollen recording obviously yielded wrong (i.e., too late) results. Overall, discrepancies between phenological flowering observations and airborne pollen measurements were common; their median across all stations and studied taxa was −15 days (even −17 days when excluding the non-reliable cases), indicating in general earlier airborne pollen than flowering onset dates.

**Table 2:** Differences (days) between start of pollen (SOS_P_) and of flowering season (SOS_F_) of seven taxa at the stations Erlangen, Munich, and Oberjoch.

	**Erlangen**							**Munich**							**Oberjoch**						
	***Corylus***	***Alnus***	***Betula***	***Fraxinus***	***Pinus***	***Poaceae***	***Artemisia***	***Corylus***	***Alnus***	***Betula***	***Fraxinus***	***Pinus***	***Poaceae***	***Artemisia***	***Corylus***	***Alnus***	***Betula***	***Fraxinus***	***Pinus***	***Poaceae***	***Artemisia***
**1987**	−11	−13	n.a.	−15	4	n.a.	n.a.	n.a.	n.a.	n.a.	n.a.	n.a.	n.a.	n.a.	n.a.	n.a.	n.a.	n.a.	n.a.	n.a.	n.a.
**1988**	57	4	n.a.	**−11**	−2	n.a.	n.a.	62	15	n.a.	**−11**	−7	n.a.	n.a.	n.a.	n.a.	n.a.	n.a.	n.a.	n.a.	n.a.
**1989**	−2	−11	n.a.	−28	−2	n.a.	n.a.	9	4	n.a.	−23	−9	n.a.	n.a.	n.a.	n.a.	n.a.	n.a.	n.a.	n.a.	n.a.
**1990**	1	−16	n.a.	−30	−6	n.a.	n.a.	3	−6	n.a.	−47	−7	n.a.	n.a.	n.a.	n.a.	n.a.	n.a.	n.a.	n.a.	n.a.
**1991**	−1	−9	−12	−35	−3	14	1	−5	−10	−12	−23	−6	1	−32	n.a.	n.a.	n.a.	n.a.	n.a.	n.a.	n.a.
**1992**	3	−11	**−10**	−21	0	1	−33	1	−5	**−13**	−14	0	13	12	n.a.	n.a.	n.a.	n.a.	n.a.	n.a.	n.a.
**1993**	22	−9	−6	−16	−7	7	−53	3	−18	−4	−5	−6	9	−15	n.a.	n.a.	n.a.	n.a.	n.a.	n.a.	n.a.
**1994**	3	−26	−16	−25	−2	4	2	1	−35	−23	−29	−4	12	−44	n.a.	n.a.	n.a.	n.a.	n.a.	n.a.	n.a.
**1995**	10	−10	−5	**−19**	−7	−4	4	n.a.	n.a.	5	5	−15	−6	4	−48	−46	−43	**−48**	−37	−21	9
**1996**	0	−6	−3	−6	0	12	−51	−3	**−10**	−5	**−10**	−2	7	−16	**−30**	**−36**	**−29**	**−30**	−23	−13	−6
**1997**	−6	−13	−15	−35	−4	2	−24	d.	d.	**−21**	−30	−24	3	11	−31	**−33**	**−52**	−68	−33	**−22**	−7
**1998**	3	−11	−15	−31	−3	1	−14	−28	−45	−15	−21	−16	−2	−6	−41	−41	−55	−54	*−21*	*−13*	−6
**1999**	**−14**	−13	**−10**	−22	−5	−4	3	**−39**	−37	**−14**	**−16**	−11	7	−19	**−84**	−68	**−48**	**−42**	−29	**−21**	−13
**2000**	−3	**−23**	−8	−26	−3	5	−58	**−16**	**−27**	−8	−15	−7	6	13	**−54**	**−48**	**−32**	−32	−21	**−15**	8
**2001**	21	1	**−18**	−46	−3	0	6	1	−15	**−22**	−24	−6	1	−4	−32	−33	**−55**	−61	−25	−17	15
**2002**	26	12	**−11**	−34	*−17*	*24*	7	**−11**	**−22**	**−15**	**−33**	−7	5	−8	**−42**	**−38**	**−56**	**−65**	*−31*	***−16***	−32
**2003**	−1	−4	d.	d.	**−12**	d.	14	−38	−45	−9	**−25**	**−12**	16	−6	*−36*	***−22***	**−33**	**−46**	−20	−12	−37
**2004**	18	−11	−9	***−14***	*−3*	13	−38	**−13**	**−34**	−35	**−21**	0	7	4	**−60**	**−55**	−34	**−50**	**−19**	**−21**	17
**2005**	3	−5	**−13**	**−17**	−6	16	−55	d.	−9	−18	**−22**	−13	2	−10	**−58**	**−23**	**−48**	**−46**	**−33**	−18	1
**2006**	2	−6	−4	**−23**	−5	0	*−7*	−4	−9	−8	−11	−8	1	*−11*	**−35**	**−23**	**−26**	**−46**	**−22**	−23	16
**2007**	**46**	2	−6	−31	−8	2	6	−12	**−35**	−8	−15	−10	0	10	−49	**−52**	−25	**−25**	**−25**	**−21**	16
**2008**	−5	−19	−6	−43	−2	3	*14*	**−16**	**−26**	−11	−20	−3	1	15	−41	**−57**	−35	−60	**−23**	−12	17
**2009**	−3	**−15**	*−30*	*−9*	*−10*	3	−30	**−11**	**−17**	−9	**−12**	**−17**	5	−43	**−38**	**−40**	**−30**	**−43**	−31	**−20**	3
**2010**	**−13**	**−21**	*−25*	***−17***	−1	*14*	*−29*	**−17**	**−21**	**−12**	**−19**	−4	*3*	−44	**−42**	**−40**	**−40**	**−34**	**−24**	−19	53
**2011**	−12	−14	−17	−10	−9	−7	5	**−15**	**−26**	**−11**	**−19**	−9	1	−9	**−46**	**−44**	**−28**	**−33**	**−32**	**−22**	4
**2012**	−20	−6	***−12***	*−29*	*0*	*1*	*11*	−1	−8	−12	**−22**	−2	5	*0*	**−35**	**−25**	**−51**	**−42**	−18	−10	−3
**2013**	d.	d.	d.	d.	d.	d.	d.	−26	−22	−9	−8	−14	3	−16	−88	**−64**	**−28**	**−26**	−33	**−15**	−21
**2014**	n.a.	n.a.	n.a.	n.a.	n.a.	n.a.	n.a.	−6	−17	−11	−27	−11	7	−29	−33	**−43**	−35	**−39**	−37	−13	−8
**2015**	n.a.	n.a.	n.a.	n.a.	n.a.	n.a.	n.a.	−22	−42	−9	**−19**	−14	7	−5	−45	**−47**	**−31**	**−34**	−28	**−14**	9
**2016**	n.a.	n.a.	n.a.	n.a.	n.a.	n.a.	n.a.	−8	−15	−8	−11	−8	5	−36	**−31**	−42	**−37**	−34	−30	−5	8
**2017**	n.a.	n.a.	n.a.	n.a.	n.a.	n.a.	n.a.	−6	**−18**	**−10**	−17	−7	5	−45	−24	**−26**	**−33**	−62	−38	−14	−15

*Likely pollen sources (light blue boxes—pre-season transport, white—local contributions, red—too late start of trap operation, and light red—other erroneous data), as well as long-range transport events (bold numbers), were classified according to rules in Table 1. n.a. missing entries (either pollen and/or phenological data), d. discarded entries (>~20% of daily data in pollen season missing), italics entries for which between 10 and 20% of daily data in the respective pollen season were missing*.

At the high elevation station Oberjoch median differences per taxa were predominantly negative, decreasing during the year from −42 days (*Corylus*) or −43 days (*Alnus*) to −18 days (*Poaceae*), only *Artemisia* pollen was ~4 days later recorded than the interpolated flowering map suggested. At the two lowland stations of Erlangen and Munich, *Corylus, Alnus, Betula*, and *Fraxinus* pollen were measured earlier than the start of flowering was monitored (median differences −11, −16, −11, and −19 days, respectively). For *Artemisia* and *Pinus*, this median difference was −9.5 and −8 days, and for grass pollen even only +3 days. For *Corylus, Alnus, Betula, Fraxinus, Pinus*, and *Poaceae* at Erlangen and Munich (2005–2012), their median annual difference of SOS_P_ and SOS_F_ varied between −5.5 days (2006) and −15 days (2010).

The classification of likely pollen sources, as well as type of transport events based on the rules listed in Table 1, revealed the following findings for the period 2005 to 2015. For all pollen taxa (except *Artemisia)* and station ^*^ year combinations, the pollen sources were non-local pre-season in 68.7%, and local in 27.9% of the cases. For six cases at Erlangen (3.4%) it had to be assumed that the pollen recording of *Corylus, Alnus*, and *Poaceae* was obviously wrong. Naturally, all cases at the high elevation station Oberjoch were non-local pre-season transport.

Concerning all cases of non-local pollen sources, 57.7% of the pre-season transport could be regarded as long-range, for 42.3% a further differentiation was not possible. At Erlangen and Munich, local pollen sources of *Pinus* and *Poaceae* were more frequent than non-local ones whereas, for the other pollen taxa, non-local sources were predominant. For *Artemisia*, the interpolated flowering dates were less reliable and thus the respective numbers were 33.3% non-local, 40.0% local sources, and 26.7% likely wrong data (4 times at Oberjoch, and twice each at Erlangen and at Munich).

These above summarized results were largely confirmed by the full picture of all data available for 1987–2017, especially at the station Oberjoch. At Erlangen, pollen monitoring seemed to start too late since *Corylus* and sometimes *Alnus* pollen was recorded later than the local flowering estimates, similarly in fewer years for Munich. For *Corylus* (only Munich), *Alnus, Betula*, and *Fraxinus* at the lowland station Erlangen and Munich, pollen was recorded consistently earlier than local flowering (median differences between −11 and −19 days). *Poaceae* pollen was observed around the mean full flowering dates of the two grass species; however, the full data set revealed more years in which pollen was even recorded much later (≥10 days). Inherent variations in the start of the *Artemisia* pollen season, probably due to low pollen concentrations, led to high variability in their match to SOS_F_ dates.

### Transportation Modeled by HYSPLIT

**Figure 4:**
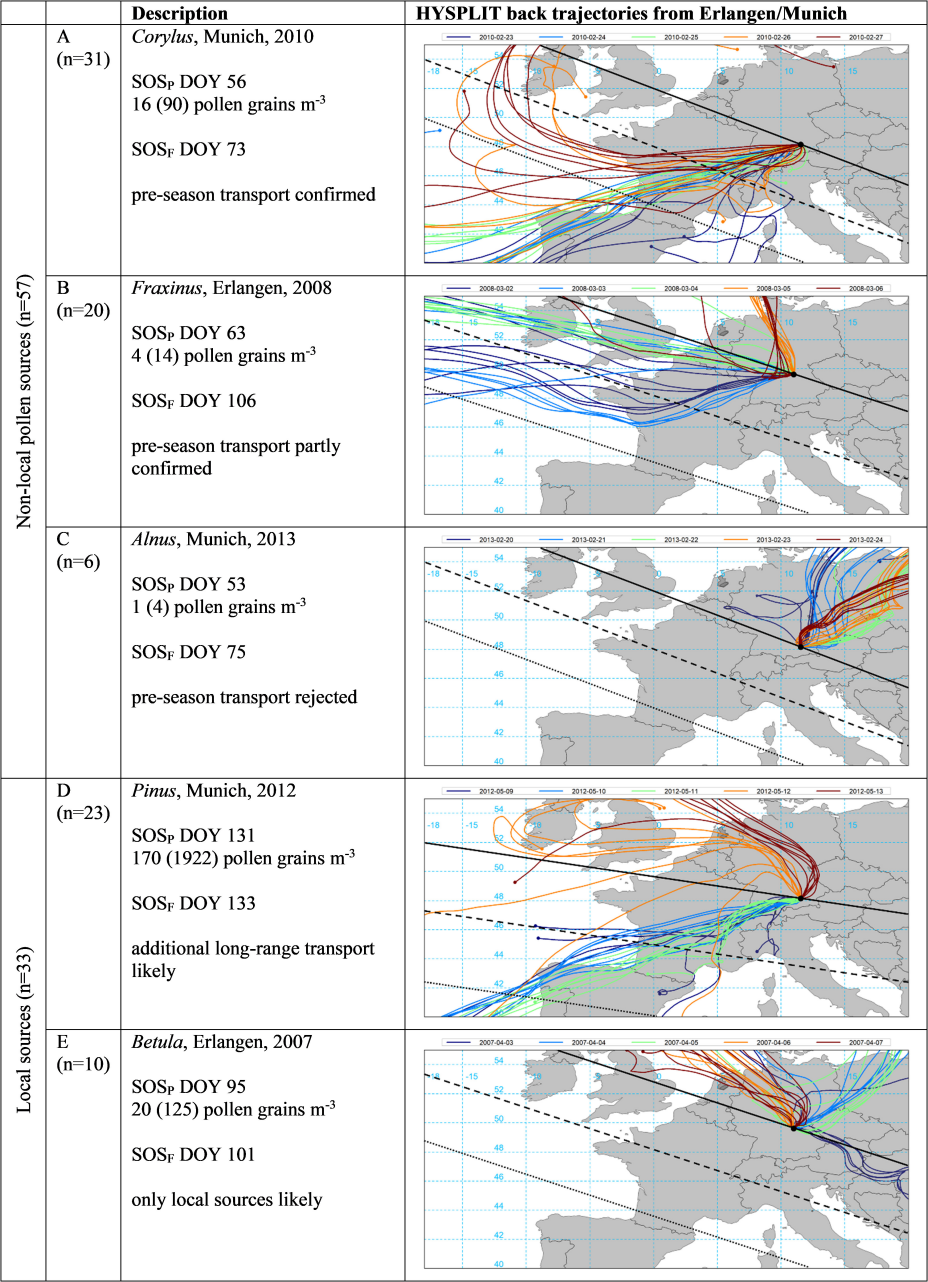
Selected examples of start of pollen (SOS_P_) in relation to start of local flowering season (SOS_F_, both in DOY) and the respective HYSPLIT back trajectories for the SOS_P_ date (green) as well as the 2 days prior and after (see section HYSPLIT Transport Modeling). Pollen concentrations are for the day of SOS_P_ as well as in brackets the summed daily concentration for the 5 days displayed. The black line indicates flowering dates identical to SOS_F_, dashed and dotted lines 10 and 20 days prior to SOS_F_, respectively, as estimated by the mean speed of the green wave according to Menzel et al. (56) (see Supplementary Table 2). Examples represent 90 cases for five pollen taxa (*Alnus, Betula, Corylus, Fraxinus*, and *Pinus*) at the stations Erlangen and Munich.

For the selected years 2005 to 2015, we tried to confirm or reject the hypothesized transport events of Table 2 based on back trajectory modeling (see section HYSPLIT Transport Modeling) and the assumptions according to possible pollen sources (see section Likelihood of Transport Based on Back Trajectories and Figure 4).

The vast majority of the studied SOS_P_ dates (in day of the year, DOY) indicated that on the same day or the 2 days before pollen was likely to be transported from areas in Europe with earlier local flowering, often even equal or earlier than SOS_P_ [see example A in Figure 4 where *Corylus* pollen was likely to be transported from the NW of the Iberian Peninsula arriving 17 days before local flowering at Munich with medium pollen loads according to the classification of (44)]. Roughly 50% of these cases might have been due to long-range transport (as indicated by similar SOS_P_ dates across Bavaria, see Table 1).

For a quantitative analysis at the lowland stations Erlangen and Munich, we omitted *Artemisia* due to too high variability in pollen data and less reliable flowering interpolation, *Poaceae* since SOS_P_ almost perfectly matched SOS_F_, and the four cases with too late *Corylus* and *Alnus* pollen monitoring at Erlangen (see Table 2, *n* = 90). In 54% of the 57 cases for non-local sources of *Alnus, Betula, Corylus, Fraxinus*, and *Pinus*, pre-season long-range transport could be confirmed by HYSPLIT back trajectories (Figure 4 example A). When SOS_P_ was much earlier than SOS_F_, back trajectories could often only indicate likely transport since flowering in the source regions (SOS_T_) was only earlier than SOS_F_ but not than SOS_P_ dates (35% of the cases, see example B where air masses originated from an area in the NW of France and SW of England with ~ 20 but not 43 days earlier flowering and transporting only small pollen loads). It was only for six out of 57 events (11%) with non-local pollen sources that a hypothesized transport could not be confirmed by back trajectory modeling [see example C where air masses came from NE directions with assumed later flowering dates and the daily pollen concentrations where according to Galán et al. (42) nil.]. In 23 of 33 cases with local pollen sources (70%), trajectories were again strongly pointing to additional pollen contributions by transport (see example D in which pollen was recorded almost the same day as local flowering, but additional long-range transport from SW France and NW Spain was very likely). In 30% (10 out of 33 cases) trajectories did not indicate any pollen transport in addition to the supposed local pollen sources [example E where air masses from the NE were unlikely to transport *Betula* pollen, daily pollen concentrations at SOS_P_ were classified as small according to Galán et al. (42), de Weger et al. (43), and Deutscher Wetterdienst (44)].

**Figure 5:**
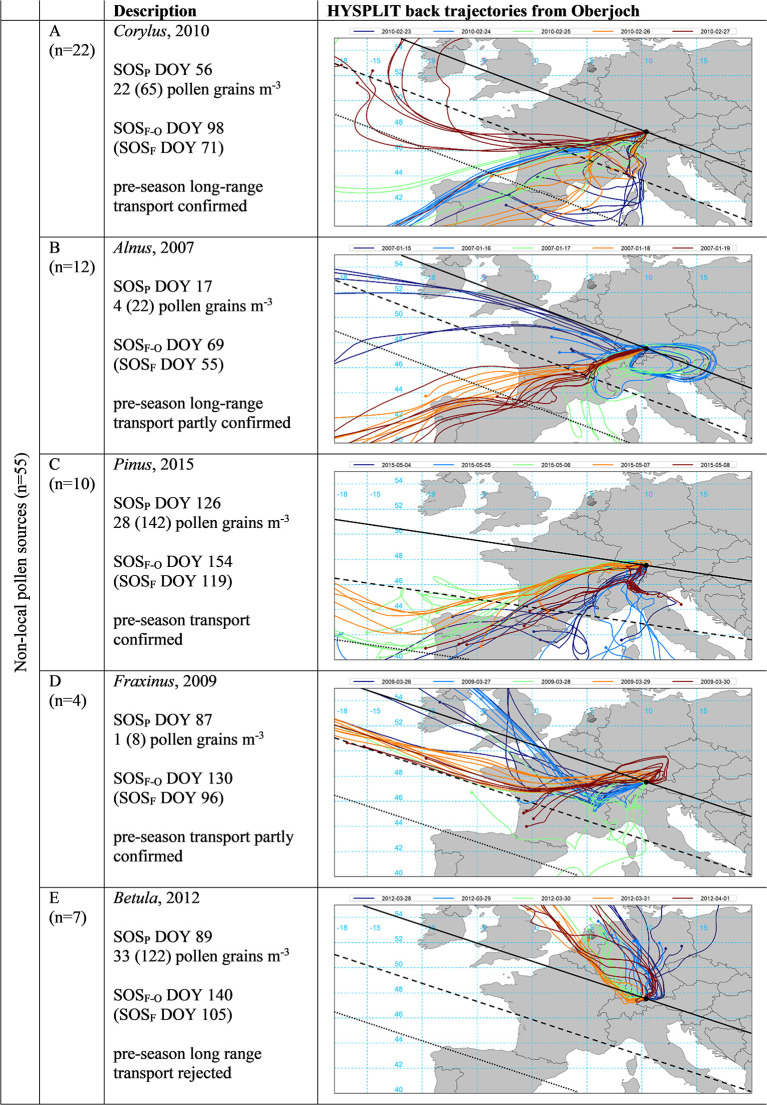
Start of pollen season (SOS_P_) in relation to start of local flowering at Oberjoch (SOS_F−O_) and at Munich / Erlangen (SOS_F_, all in DOY) and the respective HYSPLIT back trajectories for the SOS_P_ date (green) as well as the 2 days prior and after (see section HYSPLIT Transport Modeling). Pollen concentrations are for the day of SOS_P_ as well as in brackets the summed daily concentration for the 5 days displayed. The black line indicates flowering dates identical to SOS_F_, dashed and dotted lines 10 and 20 days prior to SOS_F_, as estimated by the mean speed of the green wave according to Menzel et al. (56) (see Supplementary Table 2). Examples represent 55 cases for five pollen taxa (*Alnus, Betula, Corylus, Fraxinus*, and *Pinus*), long-range transport refers to transport from outside Bavaria (see Table 1 for the classification rules).

For a quantitative validation of pollen transport to Oberjoch, we discarded *Artemisia* as well as *Poaceae* yielding 55 (5 pollen species ^*^ 11 years) events (Figure 5). Since all pollen sources were defined as pre-season non-local (SOS_P_ at least 10 days earlier than interpolated SOS_F_) (see section Pre-season Transport Events), we classified transport events into probably long-range from outside Bavaria (75%) and others. In only seven out of 55 cases (13%), it was not possible to confirm pollen transport by back trajectories (see example E in Figure 5 where air masses from the NW were unlikely to carry already *Betula* pollen). For 22 (12) of the 55 cases, pre-season long-range transport could be confirmed (partly confirmed) and for 10 (4) of the cases, pre-season transport could be confirmed (partly confirmed) by back trajectories from Oberjoch.

**Figure 6:**
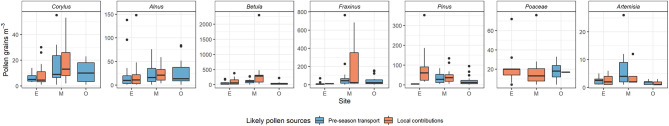
Daily pollen concentrations (pollen grains m^−3^) at the start of the pollen season (SOS_P_) for years with pre-season transport compared to years with local contributions (see Table 2 for likely pollen sources per species, year, and station).

The daily pollen concentrations at SOS_P_ in Oberjoch associated with the examples provided in Figure 5 were either above the threshold of medium pollen loads (A *Corylus*, E *Betula*) or below (B *Alnus*, D, *Fraxinus*) according to respective thresholds given by Galán et al. (42), Deutscher Wetterdienst (44), and De Weger et al. (43). Daily pollen concentrations on SOS_P_ days with pre-season transport were not significantly different from respective amounts on SOS_P_ dates with local contributions (see Figure 6) although for early season pollen species (*Corylus, Alnus, Betula*, and *Fraxinus*) the mean concentrations seemed to be slightly higher. The clinical relevance of SOS_P_ pollen concentrations generally depended on taxa and site, less on likely pollen sources (pre-season transport or local contributions). For *Corylus, Alnus*, and *Betula* SOS_P_ pollen concentrations and *Poaceae* local contributions at Munich, the threshold of medium pollen loads was reached.

## Discussion

Pre-season pollen transport proved to be a quite common phenomenon for *Alnus, Betula, Corylus, Fraxinus*, and *Pinus* in Bavaria. This could be demonstrated by different lines of evidence for the two lowland pollen stations Erlangen and Munich. In 37% of the 90 cases, SOS_P_ was in the range of SOS_F_ uncertainty, but in 63% SOS_P_ was at least 10 days earlier than SOS_F_ strongly suggesting pollen transport. For 46% of these transport events, SOS_P_ were similar across Bavaria hinting to long-range transport. For the alpine station Oberjoch, where interpolated flowering data suggested transport phenomena for all 55 cases, even 75% could be seen as from outside Bavaria.

These findings are based on a match/mismatch of SOS dates based on pollen and phenological flowering observations, as previously done e.g., by Ranta et al. (30), Estrella et al. (31), and Jochner et al. (59). Based on 40 stations and 5 years of data across Germany, Estrella et al. (31) reported that the start of the pollen season was on average 5.7 days earlier than local flowering, which is less than the mean advance for Erlangen and Munich (−11.9 days) in our study. Similarly, Ranta et al. (30) confirmed for Finland that the birch pollen season could not be determined by phenological observations alone. However, no frequency measures on pollen transport have been reported so far except Sofiev (32) who quantified for Europe that up to 20% of the inter-annual variability in the seasonal birch pollen index was due to transport, a percentage that is below our findings for two stations in Bavaria.

Therefore, the question has to be asked whether these high numbers of postulated pollen transport events are realistic. Of course, their ranges depend on the initial definition of local pollen sources or main pollen season and consequently on the accuracy of the interpolated flowering data we had to rely on. Jochner et al. (60)'s study on pollen and flowering dates of *Betula* suggested that the finer the spatial resolution of flowering observations, the higher the accuracy of flowering and pollen season match to be expected. This lack of local flowering data for the whole assumed radius of 30 km around a pollen trap may account for some inaccuracy, especially for the city of Munich. Urban heat islands are well-known for earlier flowering than their rural surroundings, e.g., in the range of 0 to 4 days for *Betula* (61) and up to 7 days for *Corylus* (62). However, in this study a threshold of even 10 days was used to account for all inherent meso- and microclimatic effects. This threshold was well above the interpolation uncertainty of flowering data across Bavaria (50). The median difference between start of the pollen season and first flowering dates of −15 days (−17 days if a few unrealistic data were excluded) allows a quick assessment of the sensitivity of this threshold definition. Even with a threshold of ±15 days for local pollen sources, 50% of the events would be related to transport.

Equally the method of how to define the start of the pollen season might be of influence. Here we used the percentage method [see (35, 36)], by which the earliest and latest annual 2.5% were discarded. Estrella et al. (31) relied on a 5% cut off, which might at least partially contribute to the fact that their mean difference between start of birch flowering and start of pollen season was smaller than in our study.

Previous findings in the literature [e.g., by (28)] have also suggested that land use information/land cover data such as the possible abundance of species may play a role. We believe that we see this abundance effect in the ranking of median flowering—pollen season differences of *Fraxinus, Alnus, Corylus, Betula, Artemisia, Pinus*, and *Poaceae* at Munich and Erlangen with −19, −16, −11, −11, −9.5, −8, and +3 days, respectively. This ranking mirrors to a certain degree the abundance of these taxa in the Bavarian landscape. For *Artemisia* also a higher inaccuracy of map interpolation (e.g., at the high altitude station flowering was even a bit earlier than in the lowlands) as well as a general high variation in their pollen season (63) might have contributed to this result. In the case of grass pollen, the phenological observations relate to the stage of full flowering (BBCH65) of two early flowering grass species instead of first flowering dates as for all other pollen taxa studied. This might also contribute to the closer match of phenological and airborne pollen data, which has been previously also reported by Estrella et al. (31).

What makes our study unique is the systematic verification of hypothesized transport events by back trajectory modeling. And this quantitative information was more than convincing. In only 13% of the studied cases for *Alnus, Betula, Corylus, Fraxinus*, and *Pinus* at Oberjoch and in 11% at the lowland stations, HYSPLIT back trajectories in combination with green wave speed and direction did not support the assumed pre-season pollen transport. When more additional higher trajectory starting heights, e.g., 1,500 or 2,000 m a.g.l. [see (15)], had been tested, pre-season transport could possibly be rejected in even fewer cases than 13 or 11%. Looking more into the cases where transport was likely, for ~ 3/5 of them pollen transport was confirmed and for ~ 2/5 only partly confirmed. This ratio might increase once more and complete flowering data at a high spatial resolution across Europe will be available, making rough assumptions via speed and direction of the green wave unnecessary. Daily pollen concentrations on SOS_P_ days were not significantly different from amounts on SOS_P_ days with local pollen contributions, but for some pollen taxa the respective amounts, especially at Munich, were classified as medium intensity. This high number of transport events is to some extent supported by Veriankaite et al. (17) who found 24 cases of birch pollen transport to three pollen stations during 4 study years in Lithuania. Future studies should also try to integrate parameters of transport definitions by Sofiev et al. (7) such as atmospheric boundary layer height, wet removal, or exchange between the boundary layer and the free troposphere into pollen transport trajectory studies.

With climate change, and mostly due to increasing atmospheric CO_2_ concentrations, pollen concentrations have mostly increased [e.g., (64)]. Like in this study, we found changes in pollen concentrations especially increases for *Corylus* and decreases at all stations for *Poaceae* and *Artemisia*, had mixed results for *Alnus, Betula, Fraxinus*, and *Pinus*. Twenty seven percentage of our trends in pollen concentrations were significant, in contrast to ~17% of the changes in start, peak and end of the pollen seasons. Especially for early spring taxa, the pollen season started by up to 2 days per year earlier, and thus it will become more and more important to start pollen monitoring in time after winter. However, by determining the start, peak and end of season pollen trends by four other methods besides the percentage method (see Figure 2), a considerable variation of strength of the resulting linear trends was obvious among methods and stations. For the early flowering species such as *Corylus* and *Alnus*, SOS_P_ by percentage method revealed stronger advancing trends, but not necessarily always the strongest. Although there is plenty of literature on how climate change is altering the pollen season [e.g., (1, 3, 4)], these rates of change are far stronger than any trend in corresponding flowering dates. Menzel et al. (65, 66) reported for the period 1971–2000 and till 2018 in Central Europe a mean advance of spring leaf unfolding and flowering of −0.25 days yr^−1^ with 54% of the time series being significant in the update period. Thus, these mean advances are only 1/10 of the maximum found for the start of the *Corylus* and *Alnus* pollen season. Also regarding the extremes, there are some indications that start of flowering season trends may not exceed pollen trends. The strongest advances of *Corylus* flowering observational dates determined for single stations in Bavaria did not exceed −0.8 days yr^−1^. Jochner et al. (60) reported for the cities of Frankfurt, Cologne and Munich with their urban heat islands a mean advance of *Corylu*s flowering of only −0.50 days yr^−1^.

Further studies would be needed to check whether longer dry spells in spring under climate change conditions would also favor successful long-range transport events. This underlines that phenological flowering observations seem by far more robust and less dependent on single year extreme events. A common explanation for this feature is that the analysis of pollen data is impaired by missing data as well as failing procedures to determine the key parameters of the pollen season in the R package AeRobiology. We believe that frequent transport events will also contribute to this lack of correspondence in pollen and flowering trends, thus deriving trends in pollen season should be much dependent on medium or long-range transport. In turn, distributional areas and abundance of relevant species, convection in the atmosphere, frequency and efficiency of atmospheric circulation patterns transporting air masses from the SW will be important factors for key parameters of the pollen season in Central Europe.

## Conclusions

Sensitization to pollen allergens is increasing in most regions of Europe and the prevalence of allergic rhinitis already amounts to up to 30% (67). Thus, timing and allergenic pollen sums are critical factors. Our first climatology of pollen transport to Bavaria highlights the importance of this phenomenon also in quantitative respect: 75% of SOS_P_ at the alpine station are from outside Bavaria, 63% of SOS_P_ at the lowland stations are related to non-local pollen sources and even in the main pollen season, and 70% of events may receive additional transported pollen on top of the local sources. This means that the actual pollen concentration is less dependent on local conditions complicating pollen forecast by pure phenological information. Since pollen transport might deliver more pollen, additional pollen during night time, pollen from alien species with other allergens, prolong the local pollen season and render it more variable at the end, more studies to further quantify pollen transport, also for climate change and/or land use-land cover change scenarios are needed.

## Data Availability Statement

The data analyzed in this study is subject to the following licenses/restrictions: The analyzed datasets for this study can be found in the databases of the German Meteorological Service (DWD, free online access) as well as of the German Pollen Information Service (Stiftung Deutscher Polleninformationsdienst, PID, available upon request). Requests to access these datasets should be directed to the German Pollen Information Service (Stiftung Deutscher Polleninformationsdienst, PID).

## Author Contributions

AM: conceptualization, funding acquisition, methodology, project administration, supervision, writing of the original draft, and review and editing. HG: data curation, formal analysis, visualization, and writing—review and editing. YY: formal analysis, visualization, and writing—review and editing. NE: methodology, project administration, supervision, and writing—review and editing. All authors contributed to the article and approved the submitted version.

## Conflict of Interest

The authors declare that the research was conducted in the absence of any commercial or financial relationships that could be construed as a potential conflict of interest.
