# Supplementary material for: A First Pre-season Pollen Transport Climatology to Bavaria, Germany
Source: Front Allergy. 2021 Feb 25;2:627863. doi: 10.3389/falgy.2021.627863 (PMC8974717; doi:10.3389/falgy.2021.627863)
Supplement: Supplementary file 1 [file Data_Sheet_1.PDF]

Supplementary Material

1 Supplementary Tables

Supplementary Table S1. Location and pollen trap height of the six pollen stations across Bavaria.

Pollen station	Latitude (°)	Longitude (°)	Altitude (m a.s.l.)	Pollen trap height (~ m a.g.l.)
Münnerstadt	50.2506	10.1781	347	15
Bamberg	49.9011	10.8856	238	11
Erlangen	49.6000	11.0075	284	10
Zusmarshausen	48.4044	10.6119	483	10
Munich	48.1289	11.5644	538	20
Oberjoch	47.5169	10.4037	870	06

Supplementary Table S2. Speed and direction of the green wave across Europe.

Significant multiple regression coefficients [days deg^{-1} latitude/longitude] of mean seasonal onset dates according to Menzel et al. (2005) (model with highest R^2 selected)

Season / pollen taxa	days / °latitude	days / °longitude
1: Early spring / *Corylus, Alnus*	2.4	0.8
4: Late spring I / *Betula, Fraxinus*	2.2	0.6
5: Late spring III / *Pinus*	2.2	0.3
6: Early summer / *Poaceae*	1.7	0

2 Supplementary Figures

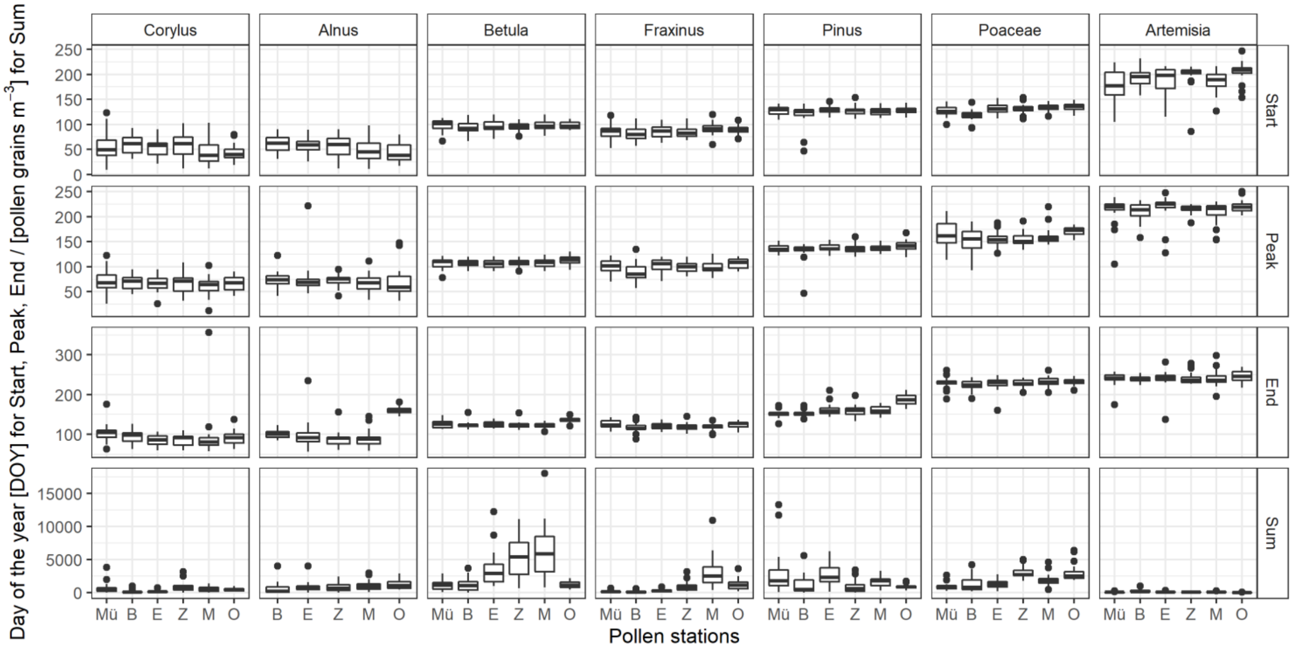

Supplementary Figure S1. Basic overview on pollen amounts at the start, peak, and end of pollen season dates as well as annual pollen sums at the six pollen stations of our study according to the percentage method (see section 2.1).

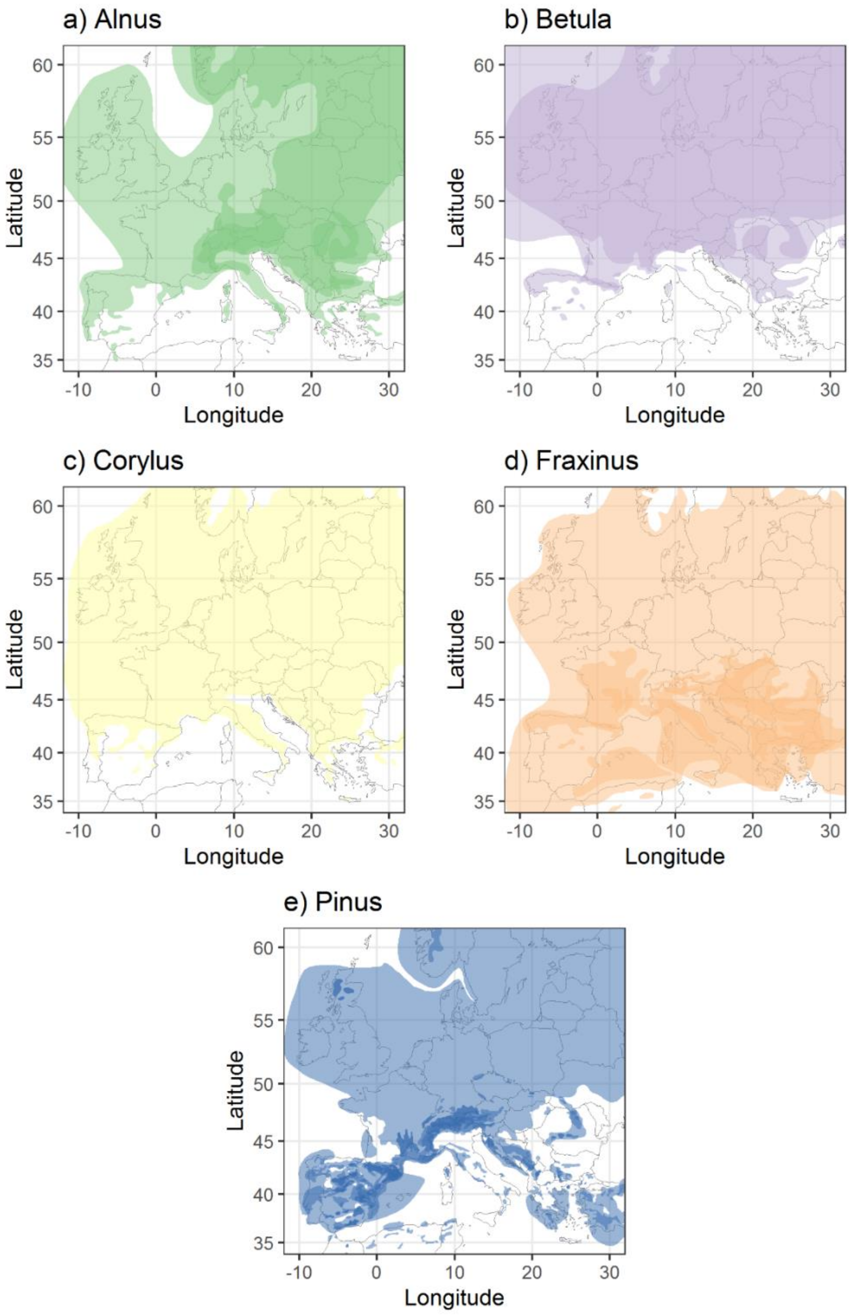

Supplementary Figure S2. Maps of taxa distribution (all available taxa assembled) in Europe for genera of a) *Alnus* (*A. glutinosa*, *A. incana*, *A. cordata*); b) *Betula* (*B. pendula*, *B. pubescens*); c) *Corylus avellana*; d) *Fraxinus* (*F. angustifolia*, *F. excelsior*, *F. ornus*); e) *Pinus* (*P. brutia*, *P. cembra*, *P. halapensis*, *P mugo*, *P. nigra*, *P. pinaster*, *P. pinea*, *P. sylvestris*). Distribution datasets were derived from research data in Caudullo et al. (2017). Coastlines were derived from Natural Earth 1:50m Cultural Vectors Admin 0 – Countries version 4.1.0 (https://www.naturalearthdata.com). The European distribution for grass species can be referenced in the grass pollen production map from Figure 1 (right) in Sofiev (2017), which shows a pan-European distribution. A. vulgaris alone (as one of the many Artemisia species) already exhibits a nearly complete European distribution based on Invasive Species Compendium of CABI (https://www.cabi.org/isc/datasheet/7108#REF-DDB-55157) and Holm et al. (1991).

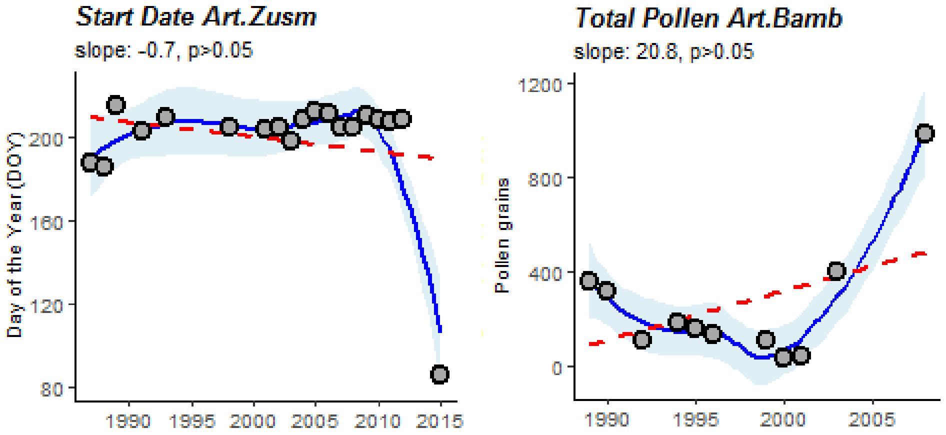

Supplementary Figure S3. Time series of start of *Artemisia* pollen season at Zusmarshausen and of annual *Artemisia* pollen sum at Bamberg.

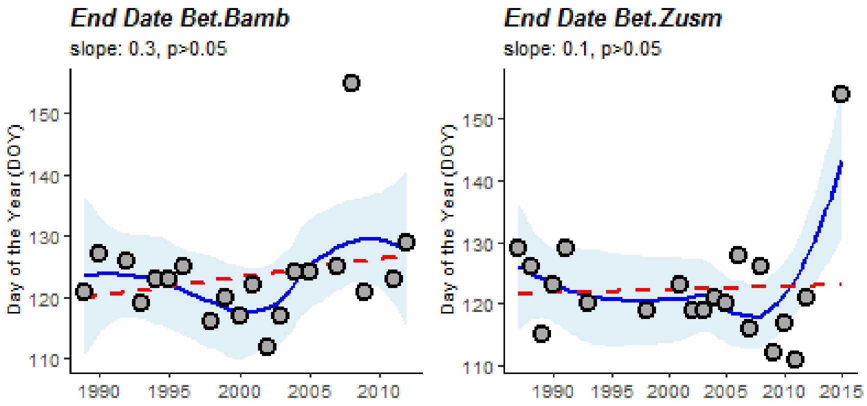

Supplementary Figure S4. Time series of end of the *Betula* pollen season at Bamberg and Zusmarshausen.

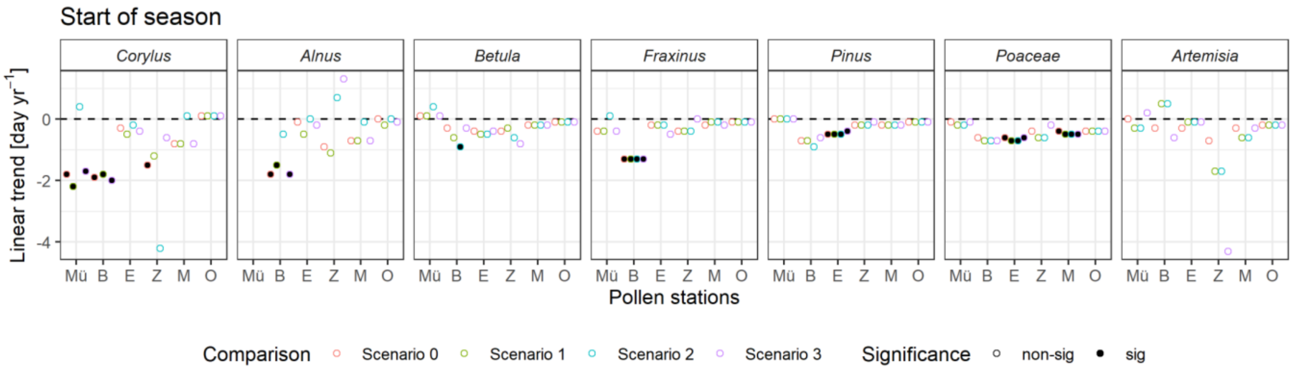

Supplementary Figure S5. Start of the pollen season changes for 7 selected taxa (column ordered by season from left to right) over 1987-2017 at 6 stations in Bavaria (from north to south Mü Münnerstadt, B Bamberg, E Erlangen, Z Zusmarshausen, M Munich, O Oberjoch). Values are slopes of linear regressions (days yr^{-1}), significance tested at the $p<0.05$ level. Scenario 0 corresponds to the percentage method as displayed in Figure 2 with all data included. Two further scenarios account for a possible too late operation start of the pollen trap in spring (see section 2.1). In Scenario 1 SOS_P dates were discarded if the first day of operation already had pollen amounts larger than 4 grains m^{-3}. In Scenario 2 SOS_P dates were discarded if the first day of operation was less than 10 days earlier than local flowering (see section 2.2). Scenario 3 is based on raw data only, thus as compared to Scenario 0 without imputation of missing data in the pollen season (see section 2.1).
